# Genome-wide investigation of *SQUAMOSA promoter binding protein-like* genes in *Liriodendron* and functional characterization of *LcSPL2*

**DOI:** 10.1093/aobpla/plae008

**Published:** 2024-02-20

**Authors:** Yu Zhang, Qinghua Hu, Xinyu Zhai, Zhonghua Tu, Jing Wang, Minxin Wang, Huogen Li

**Affiliations:** State Key Laboratory of Tree Genetics and Breeding, Co-Innovation Center for Sustainable Forestry in Southern China, Nanjing Forestry University, Nanjing 210037, China; State Key Laboratory of Tree Genetics and Breeding, Co-Innovation Center for Sustainable Forestry in Southern China, Nanjing Forestry University, Nanjing 210037, China; State Key Laboratory of Tree Genetics and Breeding, Co-Innovation Center for Sustainable Forestry in Southern China, Nanjing Forestry University, Nanjing 210037, China; State Key Laboratory of Tree Genetics and Breeding, Co-Innovation Center for Sustainable Forestry in Southern China, Nanjing Forestry University, Nanjing 210037, China; State Key Laboratory of Tree Genetics and Breeding, Co-Innovation Center for Sustainable Forestry in Southern China, Nanjing Forestry University, Nanjing 210037, China; State Key Laboratory of Tree Genetics and Breeding, Co-Innovation Center for Sustainable Forestry in Southern China, Nanjing Forestry University, Nanjing 210037, China; State Key Laboratory of Tree Genetics and Breeding, Co-Innovation Center for Sustainable Forestry in Southern China, Nanjing Forestry University, Nanjing 210037, China

**Keywords:** Flowering time, genetic transformation, *Liriodendron chinense*, SPL transcription factors family

## Abstract

The plant-specific SQUAMOSA promoter-binding protein-like (SPL) transcription factors play a pivotal role in various developmental processes, including leaf morphogenesis and vegetative to reproductive phase transition. *Liriodendron chinense* and *Liriodendron tulipifera* are widely used in landscaping due to their tulip-like flowers and peculiar leaves. However, the *SPL* gene family in *Liriodendron* has not been identified and systematically characterized. We systematically identified and characterized the *SPL* family members in *Liriodendron*, including phylogeny, gene structure and syntenic analyses. Subsequently, we quantified the expression patterns of *LcSPLs* across various tissue sites through transcription-quantitative polymerase chain reaction (RT-qPCR) assays and identified the target gene, *LcSPL2*. Finally, we characterized the functions of *LcSPL2* via ectopic transformation. Altogether, 17 *LcSPL* and 18 *LtSPL* genes were genome-widely identified in *L. chinense* and *L. tulipifera*, respectively. All the 35 *SPLs* were grouped into 9 clades. Both species had three *SPL* gene pairs arising from segmental duplication events, and the *LcSPLs* displayed high collinearity with the *L. tulipifera* genome. RT-qPCR assays showed that *SPL* genes were differentially expressed in different tissues, especially. Because *LcSPL2* is highly expressed in pistils and leaves, it was selected to describe the *SPL* gene family of *L. chinense* by ectopic expression. We showed that overexpression of *LcSPL2* in *Arabidopsis thaliana* resulted in earlier flowering and fewer rosette leaves. Moreover, we observed that overexpression of *LcSPL2* in *A. thaliana* up-regulated the expression levels of four genes related to flower development. This study identified *SPL* genes in *Liriodendron* and characterized the function of *LcSPL2* in advancing flower development.

## Teaser

In this study, we systematically analysed *SQUAMOSA promoter binding protein-like* (*SPL* ) genes in the genus *Liriodendron* , comparing 35 *SPL* genes within the genus. This analysis included a phylogenetic tree, SBP domain sequence and conserved motif analysis to reveal the evolutionary relationships of *SPLs* in *Liriodendron* . Homology analysis indicated high homology of *SPLs* between *Liriodendron chinense* and *Liriodendron tulipifera* species. After examining the expression patterns of 17 *LcSPLs* across various tissues of *L. chinense* , we selected *LcSPL2* for functional studies. Overexpression of *LcSPL2* in *Arabidopsis thaliana* accelerated flowering under long-day conditions.

## Introduction

Transcription factors (TFs) play crucial roles in plant growth and development. TFs from diverse families, such as HOMEODOMAIN-LEUCINE ZIPPER (HD-Zip), R2R3-MYB, AP2/ERF, MADS-box and SQUAMOSA promoter-binding protein-like (SPL), have been identified and documented in previous studies (Fu *et al.* 2012; Liu *et al.* 2021; Yang *et al.* 2021*b*; Zong *et al.* 2021; Tu *et al.* 2022). These TFs are recognized for their involvement in leaf and flower development. SPL proteins constitute a class of plant-specific TFs characterized by a highly conserved SBP domain consisting of 76 amino acid residues (Birkenbihl *et al.* 2005). This domain contains two zinc finger structures, one being C3H and the other being C2HC, along with a nuclear localization signal (NLS) (Klein *et al.* 1996; Yamasaki *et al.* 2004; Birkenbihl *et al.* 2005). Notably, the second zinc finger partially overlaps with the NLS, located at the C-terminal of the SBP domain, guiding SPL proteins into the nucleus to regulate the expression of target genes (Birkenbihl *et al.* 2005; Yamasaki *et al.* 2006). Among the first two SBP domain proteins reported in *Antirrhinum majus*, *AmSBP1* and *AmSBP2* were found to bind to the promoter of *SQUAMOSA*, thereby regulating early flower development (Klein *et al.* 1996). Since then, SPL proteins have been extensively identified and characterized in various plant species, including *Arabidopsis thaliana*, rice (*Oryza sativa*), poplar (*Populus trichocarpa*), maize (*Zea mays*), *Medicago truncatula*, *Codonopsis pilosula*, *Populus euphratica* and *Carya illinoinensis* (Moreno *et al.* 1997; Cardon *et al.* 1999; Xie *et al.* 2006; Li and Lu 2014; Wang *et al.* 2019, 2021; Yang *et al.* 2021*a*).

*SPLs* have been extensively studied in both model and non-model plants, underscoring their pivotal regulatory roles in various aspects of plant growth and development. These roles encompass controlling plastochron length, orchestrating the transition from vegetative to reproductive phases, determining the timing of flowering, regulating plant fertility, influencing trichome distribution, modulating lateral root development, governing floral organ development and shaping leaf morphology (Wang *et al.* 2008, 2009, 2016; Yamaguchi *et al.* 2009; Xing *et al.* 2010; Yu *et al.* 2010, 2015; Jung *et al.* 2012, 2016; Gao *et al.* 2018*a*; Cao *et al.* 2019). Furthermore, *SPLs* can be categorized into two groups: microRNA-targeted *SPLs* and non-microRNA-targeted *SPLs* (Xu *et al.* 2016; He *et al.* 2018; Zheng *et al.* 2019). Among them, microRNA156 has emerged as a major regulatory factor in the vegetative phase transitions of *A. thaliana* and other flowering plants, establishing the miR156-*SPL* pathway as a novel mechanism for age-dependent flowering regulation (Bergonzi *et al.* 2013; He *et al.* 2018). Previous studies have shown that miR156 levels are higher during the juvenile phase and significantly decrease in the adult phase, leading to an age-dependent up-regulation of *SPL* expression levels targeted by miR156. This up-regulation initiates the transition to the reproductive stage (Wang *et al.* 2009; Wang 2014). Crucially, the overexpression of miR156 negatively regulates several *SPL* genes and postpones the transition from the vegetative to reproductive stages, indicating that *SPLs* act as ageing genes, and *SPLs* overexpression promotes the transition from juvenile to adult plants (Shalom *et al.* 2015; He *et al.* 2018; De Paola *et al.* 2023). However, the functions of *SPLs* and the miR156*-SPL* module in *Liriodendron* plants have not been fully explored and remain unknown. Therefore, further research is necessary to investigate their roles in the regulation of growth and development in *Liriodendron* plants.

The *Liriodendron* genus, belonging to the Magnoliaceae family, comprises two species such as *L. chinense* and *L. tulipifera* (Chen *et al.* 2018). Both species are renowned for their exceptional wood quality and captivating appearance, particularly their tulip-like flowers. These flowers not only bestow upon them high ornamental value but also play a crucial role in their reproduction. However, the extensive geographical distribution of these species results in variations in their flowering times (Hao *et al.* 1995; Li *et al.* 2007), and the prolonged juvenile phase hinders the process of genetic improvement and their widespread utilization. Therefore, investigating the mechanism of flower induction and development, and abbreviating the juvenile phase in *Liriodendron* is of paramount importance.

In this study, we identified 17 *LcSPL* and 18 *LtSPL* genes based on the reference genomes of *L. chinense* (Chen *et al.* 2018) and *L. tulipifera* (YP108A v1.1, DOE-JGI, http://phytozome-next.jgi.doe.gov/). A comprehensive analysis was performed on these genes, including an investigation of their sequence composition, gene structure, *cis*-acting elements and collinearity. Additionally, we examined the evolutionary relationships of *LcSPL* genes across several species, including *L. chinense*, *L. tulipifera*, *M. truncatula*, poplar, *A. thaliana* and rice. Furthermore, the tissue expression profiles of *LcSPLs* in various tissues of *L. chinense* were investigated. To gain insights into the functional role of *LcSPL2* in flower development, we overexpressed *LcSPL2* in *A. thaliana* and analysed the phenotypes of the transgenic plants. These findings provide a solid foundation for further characterizing the *SPL* genes in *L. chinense*.

## Materials and Methods

### Plant materials

Samples of root, stem, leaf, sepal, flower bud, petal, pistil and stamen from *L. chinense* were collected from a provenance plantation in Jurong County, Zhenjiang City, Jiangsu Province (32°7ʹN, 119°13ʹE). Each sample was replicated three times for robustness.

For the *A. thaliana* experiments, both wild-type (WT) and transgenic *A. thaliana* plants of the Colombia ecotype (Col-0) background were used. The seeds were sown on 1/2 MS solid medium and 1/2 MS solid medium supplemented with 30 mg/L Hygromycin B, respectively, and then placed in the dark at 4 °C for 48 h. Subsequently, the seedlings were transferred to an artificial illumination incubator set at long-day conditions (16 h of light, 8 h of dark) with a temperature of 23 °C, humidity of 70 % and a light intensity of 130–150 μmol m^−2^ s^−1^.

### Identification and physicochemical properties analysis of LcSPL proteins

To identify SPL proteins from the whole genome of *L. chinense* and *L. tulipifera*, first, 16 *A. thaliana* SPL proteins (downloaded from the Plant Transcription Factor Database) were used to query sequences to blast the *L. chinense* and *L. tulipifera* protein data by performing a local BLASTP alignment with an *E* value of 1 × 10^−5^ (Chen *et al.* 2018). Then we used the hidden Markov Model (HMM) of SBP domain (PF03110) (downloaded from Pfam database (http://pfam.xfam.org/family/pf03110) to align the two types protein data by using the HMMER software (Potter *et al.* 2018)). The two types of proteins that appeared in both BLASTP alignment and HMM search results were considered as candidate LcSPL and LtSPL proteins. These SPL proteins were aligned to the Pfam database to remove proteins without the SBP domain, and the remaining proteins were identified as LcSPL proteins. Then, we used the ProtParam tool (https://web.expasy.org/protparam/) to analyse the physicochemical properties of these LcSPLs and LtSPLs.

### Phylogenetic analysis, multiple sequence alignments and conserved motif identification of LcSPL proteins

Phylogenetic trees comparing *L. chinense*, *L. tulipifera*, *M. truncatula*, popular, *A. thaliana* and rice (Cardon *et al.* 1999; Xie *et al.* 2006; Li and Lu 2014; Wang *et al.* 2019) were constructed with the Neighbor-Joining (NJ) method, and the specific parameters were Poisson model and 1000 bootstrap replications by using the MEGA software (Hall 2013). Then, we used the MEME tool (https://meme-suite.org/meme/tools/meme) to identify conserved motifs in LcSPL and LtSPL proteins (the number of conserved motifs was set as 10), and sequences of identified conserved motifs were aligned to Pfam database to confirm their corresponding domains.

### Synteny analysis, chromosome localization and *cis*-regulatory elements identification of *LcSPL* genes

Intraspecific synteny and interspecific synteny analysis were performed through the methods described in our previous study (Tu *et al.* 2022). The genome data of *A. thaliana* and poplar that were used for interspecific synteny analysis were downloaded from Phytozome 13 database (https://phytozome-next.jgi.doe.gov/). The synteny analysis results were visualized by using the Advanced Circos tool.

To identify *cis*-elements, we analysed the 2000-bp sequences upstream of the translation start site for each *LcSPLs* and *LtSPLs*. Utilizing the PlantCare online tool (http://bioinformatics.psb.ugent.be/webtools/plantcare/html/) with default parameters, we identified predicted *cis*-regulatory elements present in the gene promoters. For the determination of the gene structure and chromosome localization of *LcSPLs* and *LtSPLs*, a local BLASTN search was performed. In this search, the sequences of *LcSPL*s and *LtSPLs* were aligned to the genomes of *L. chinense* and *L. tulipifera*.

### RNA extraction and detection of gene expression level

The RNA Prep Pure Plant Kit (Tiangen, Beijing, China) and Evo M-MLV RT Premix for qPCR (AG11706, Accurate Biotechnology, Hunan Co, Ltd.) were employed for the extraction of total RNA from *L. chinense* samples and subsequent cDNA synthesis, following the respective instructions. Total RNA from WT *A. thaliana* and transgenic plants was extracted using the KK Fast Plant Total RNA Kit (ZP405K, ZOMANBIO, Beijing). Subsequently, the Evo M-MLV RT Premix for RT-qPCR was utilized for cDNA synthesis.

We used RT-qPCR to detect the expression level of *LcSPLs*. A total of 10 μL reaction volume included 10 μM forward primer, 10 μM reverse primer, 0.2 μL 50× ROX, 5 μL 2× SYBR^®^ Green Pro *Taq* HS Premix (provided by SYBR^®^ Green Premix Pro Taq HS qPCR Kit (AG11701, ACCURATE BIOTECHNOLOGY)), 1 μL cDNA and up to 10 μL RNase-free water. For the amplification process and method of data analysis, refer to our previous study (Tu *et al.* 2022). *LcACT97* was used as internal control gene for *L. chinense*, and *AtACT2* was used as a reference gene for *A. thaliana*.

### Ectopic transformation of *LcSPL2* in *A. thaliana* and phenotypic analyses

For constructing the *LcSPL2* overexpression vector, the coding sequence (CDS) of *LcSPL2* was inserted into the pCAMBIA 1300 vector at its cleavage sites using KpnI and XbaI restriction endonucleases (TaKaRa, Dalian, China). The recombinant plasmids, confirmed by sequencing, were transferred to *Agrobacterium tumefaciens* strain GV3101 through a heat-shock method and then the *Agrobacterium*-mediated floral dip method was used for the *A. thaliana* genetic transformation. T3 generation homozygous transgenic lines were selected for further analyses. The initial flowering time and the number of rosette leaves of both WT and transgenic *A. thaliana* plants were recorded. Subsequently, samples were collected from both WT and transgenic *A. thaliana* plants when the transgenic *A. thaliana* first blossomed. Each line consisted of at least 10 seedlings for phenotype observation. All the samples were immediately frozen and stored at −80 °C until use.

RT-qPCR was used to quantitatively assess the transcription levels of *LcSPLs* in various tissues of *L. chinense*. Additionally, the expression levels of flower development-related genes (*AtSOC1*, *AtFT*, *AtFUL* and *AtAP1*) were analysed in transgenic *A. thaliana*. *LcActin97* was utilized as the reference gene for *L. chinense*, while *AtActin2* served as the reference gene for *A. thaliana*. All primer sequences are provided in Supplementary Material 2.

### Data analysis

We used the 2^−ΔΔCT^ method to calculate the relative expression levels of *LcSPLs*. All RT-qPCR data were analysed with IBM SPSS Statistics (Version 25; SPSS, Inc., Chicago, IL, USA) and GraphPad Prism (version 8.0.0 for Windows, GraphPad Software, San Diego, CA, USA), the statistical validity was analysed by one-way analysis of variance (ANOVA). Duncan’s multiple range test was applied. Differences were considered significant when *P* < 0.01 or *P* < 0.05. All heatmaps were constructed with the TBtools software (https://github.com/CJ-Chen/TBtools/releases).

## Results

### Identification of *SPL* genes in *L. chinense* and *L. tulipifera*

In this study, a total of 35 *SPL* genes were identified in the genus *Liriodendron*. Specifically, 17 *SPLs* in *L. chinense* were denoted as *LcSPL1a* to *LcSPL15* (Table 1), while the 18 *SPLs* in *L. tulipifera* were designated as *LtSPL1a* to *LtSPL15* (Table 2) based on their closest orthologs in *A. thaliana*. The predicted LcSPL and LtSPL proteins exhibited considerable variability in terms of molecular weight and length. Furthermore, Table 1 provides detailed characteristics of each individual *SPL* gene, including isoelectric point (pI) and the number of predicted exons.

**Table 1.** Basic information about *LcSPLs*

Gene	CDS length (bp)	Protein length (aa)	pI	Mw (Da)	Exon number
*LcSPL1a*	3618	1205	6.32	133 382.9	10
*LcSPL1b*	3111	1036	6.21	116 154.9	10
*LcSPL2*	1395	464	7.22	33 856.09	4
*LcSPL4*	615	204	9.44	22 550.30	3
*LcSPL5*	591	196	7.13	22 603.46	2
*LcSPL7*	2610	869	6.10	97 597.23	12
*LcSPL8a*	993	330	8.88	36 836.73	3
*LcSPL8b*	1242	413	8.47	45 771.72	3
*LcSPL9*	999	332	9.50	35 359.56	3
*LcSPL10*	1128	375	7.28	41 154.83	3
*LcSPL11*	1416	471	8.42	51 996.08	4
*LcSPL12*	3195	1064	6.93	118 214.00	10
*LcSPL13a*	1233	410	8.65	45 047.38	3
*LcSPL13b*	972	323	8.54	35 021.90	3
*LcSPL14a*	3312	1103	7.71	121 827.50	10
*LcSPL14b*	3276	1091	7.22	119 699.90	10
*LcSPL15*	1218	405	9.29	42 405.11	3

**Table 2.** Basic information about *LtSPLs*

ID	CDS length (bp)	Protein length (aa)	pI	Mw (Da)	Exon Number
*LtSPL1a*	3183	1060	6.44	117 069.7	10
*LtSPL1b*	3105	1034	6.31	116 299.1	10
*LtSPL2*	1395	464	8.37	51 013.93	4
*LtSPL4*	732	243	9.33	270 88.74	2
*LtSPL5*	591	196	7.6	22 530.4	2
*LtSPL6*	1632	543	8.37	59 540.47	3
*LtSPL7*	2397	798	6.25	89 587.14	10
*LtSPL8a*	963	320	8.62	35 788.5	3
*LtSPL8b*	1242	413	8.04	45 581.43	3
*LtSPL9*	1146	381	9.31	40 643.3	3
*LtSPL10*	1116	371	7.28	40 812.52	3
*LtSPL11*	1419	472	8.42	52 124.21	4
*LtSPL12*	3183	1060	6.3	117 709.2	11
*LtSPL13a*	1233	410	8.65	45 047.44	3
*LtSPL13b*	972	323	8.34	35 006.89	3
*LtSPL14a*	3312	1103	7.34	121 956.5	10
*LtSPL14b*	3273	1090	7.46	119 591.9	10
*LtSPL15*	1218	405	9.2	42 379.02	3

### Phylogenetic analysis of the LcSPLs and LtSPLs

**Figure 1.**
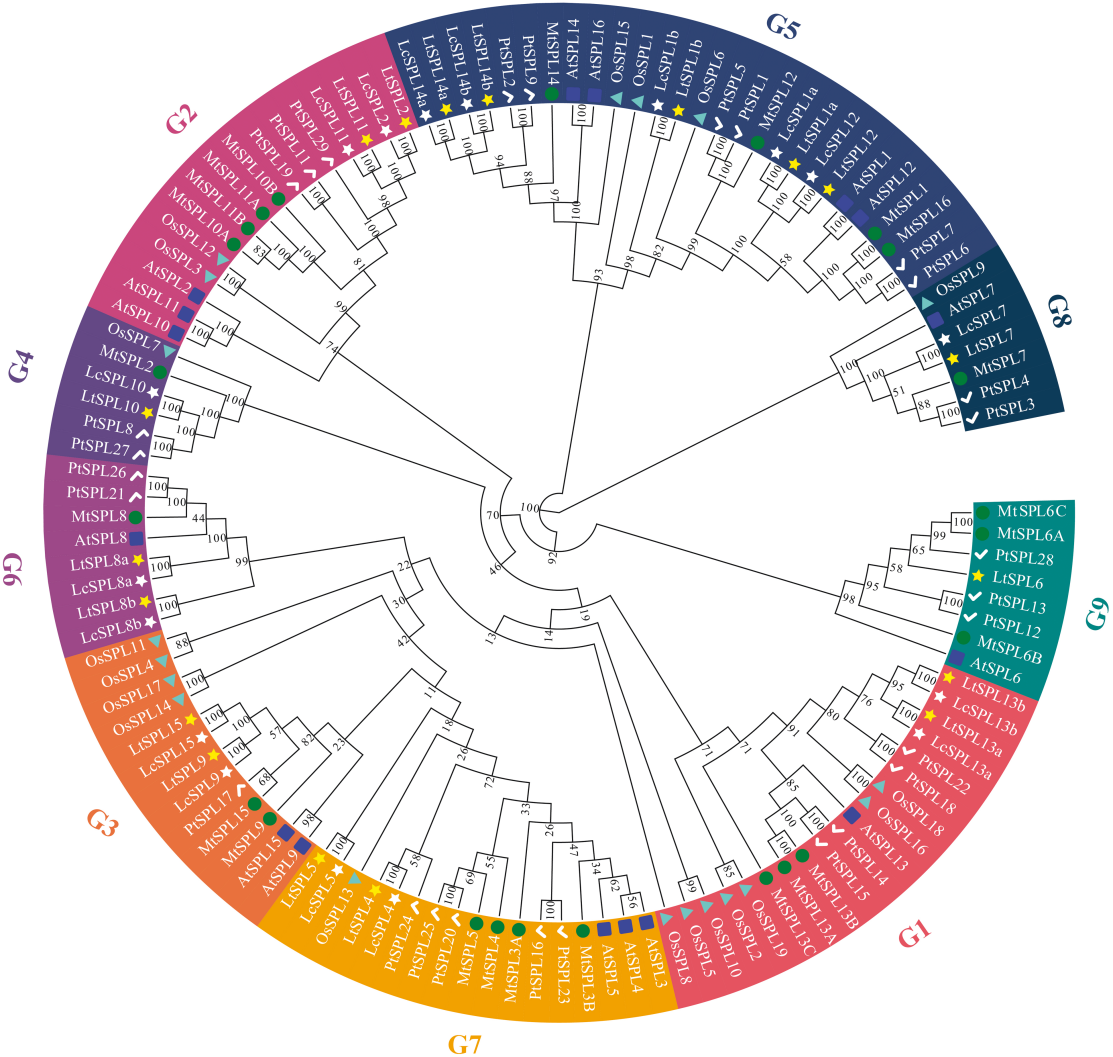
Phylogenetic tree of SPL proteins in *L. chinense*, *L. tulipifera*, *M. truncatula*, *P. trichocarpa*, *A. thaliana* and *O. sativa*. The full-length amino acid sequences of 17 LcSPLs, 18 LtSPLs, 23 MtSPLs, 28 PtSPLs, 16 AtSPLs and 19 OsSPLs were used to construct a phylogenetic tree with 1000 bootstrap replicates by MEGA11.

To investigate the evolutionary relationships between SPL proteins in *L. chinense* and other plant species, we constructed an NJ phylogenetic tree based on full-length SPL protein sequences from six species, including *L. chinense* (17), *L. tulipifera* (18), *M. truncatula* (23), poplar (28), *A. thaliana* (16) and rice (19) (Fig. 1).

In the phylogenetic tree, the 121 SPLs were classified into 9 clades, denoted as G1–G9. Notably, the number of SPLs varied among the clades, with the G5 clade containing the highest number of SPLs from *Liriodendron*, while the other 8 clades consisted of 1–2 SPLs each. It is interesting to observe that each member of the LtSPLs, except for LtSPL6, had a homologous counterpart in the LcSPLs. Within the G2 clade, SPL proteins from the same species clustered together in clades, indicating higher conservation of SPLs within species. Additionally, the G4 and G6 clades contained fewer species compared with the remaining clades, and AtSPLs were not found in the G4 clade.

### Gene structure, conserved domains and motifs of the *LcSPLs* and *LtSPLs*

**Figure 2.**
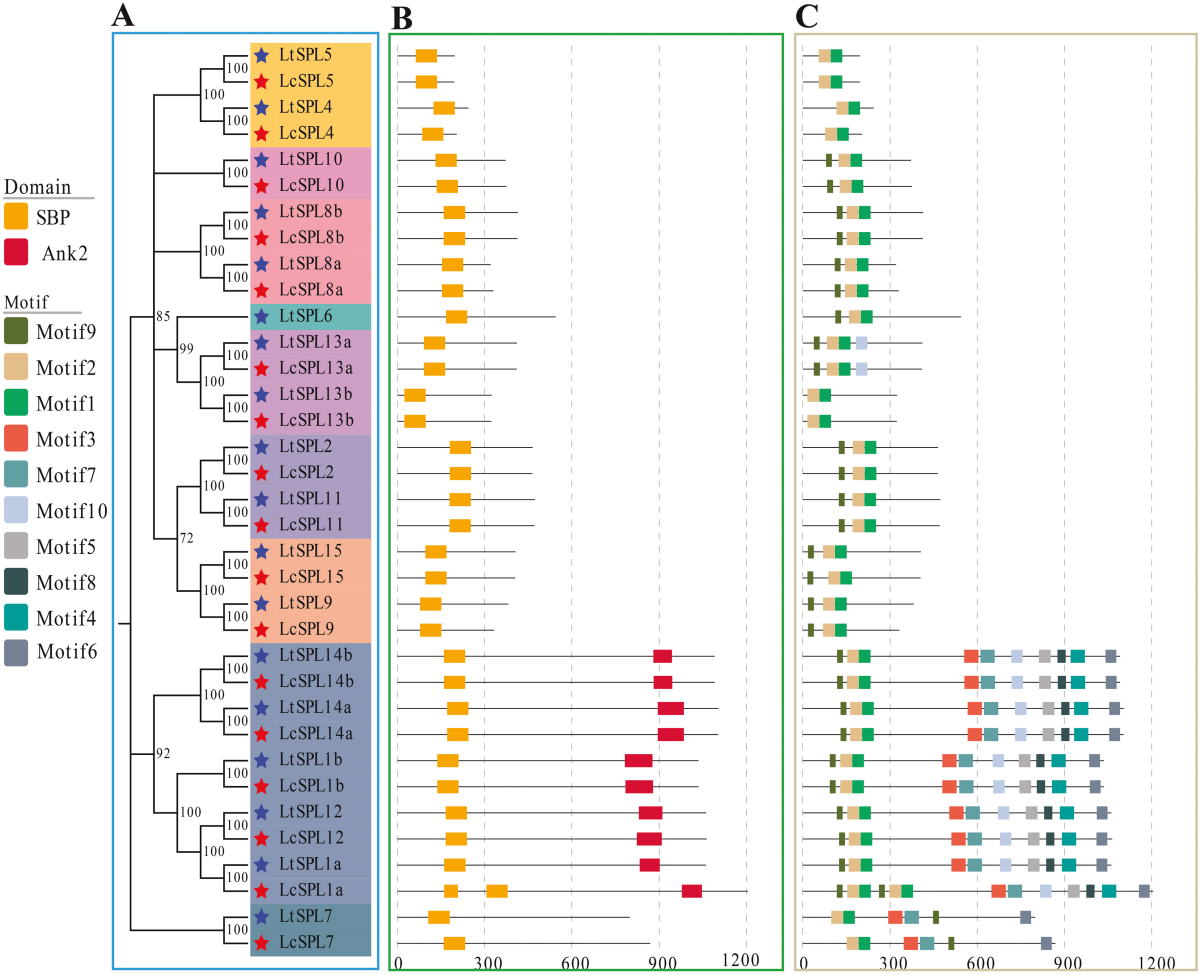
Phylogenetic relationships and architecture of the conserved motifs in SPL proteins from *Liriodendron*. (A) The phylogenetic tree was constructed based on the full-length sequences of LcSPL and LtSPL proteins. (B) Conserved domains structure of *LcSPL* and *LtSPL* genes. Black lines indicate relative protein lengths, boxes represent conserved domains and domains are colour-coded. (C) Amino acid motifs in the LcSPL and LtSPL proteins are represented by coloured boxes. Black lines indicate relative protein lengths.

To gain insights into the sequence characteristics of *SPL* genes in *Liriodendron*, we conducted a systematic analysis of gene structure, conserved domains and motifs. The gene structure analysis revealed that genes within the same evolutionary clade shared an identical number of exons (Tables 1 and 2). Similarly, SPL proteins within the same clade exhibited conserved structural domains and motifs (Fig. 2). Notably, the G5 clade displayed a more intricate structure compared with other clades, containing an additional conserved structural domain, Ank2, along with motifs 4, 5 and 8, which were unique to G5.

**Figure 3.**
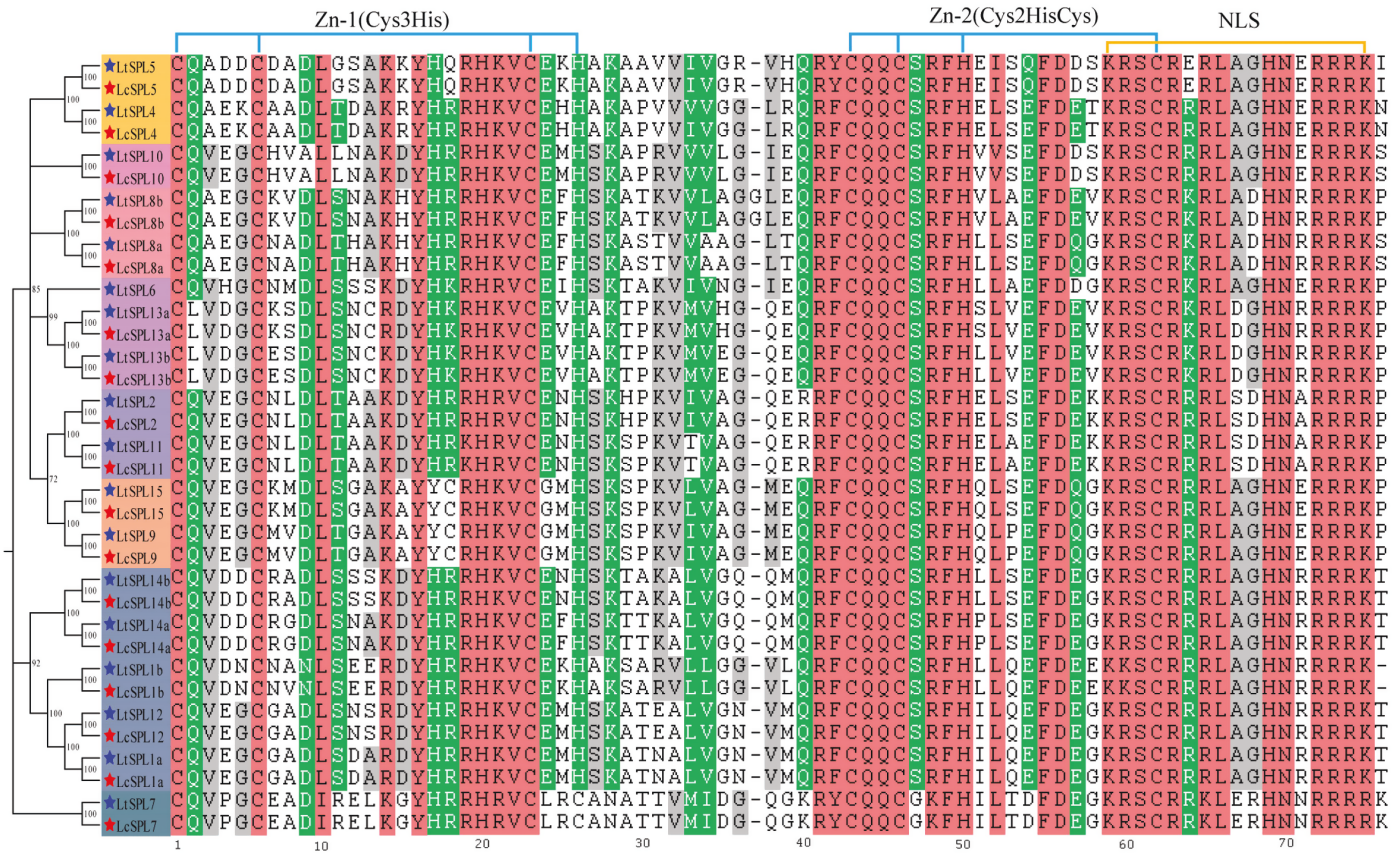
Multiple sequence alignment of SBP domain between LcSPLs and LtSPLs proteins. C3H and C2HC were two zinc finger structures, NLS was bidirectional nuclear localization signal, which were corresponding to the sequence logos of conserved domains.

Upon further comparison, it has been determined that all SPL proteins shared the SBP structural domain, comprising two zinc finger structures (Zn-1 and Zn-2) and an NLS. However, specific variations were noted at specific sites of the SBP domain (Fig. 3). For instance, the L residue was exclusively identified at the second site of the SBP domain in G1 members (LcSPL13a and LtSPL13a, LcSPL13b and LtSPL13b). In contrast, the 17th and 18th sites of the SBP domain in G3 members (LcSPL9 and LtSPL9, LcSPL15 and LtSPL15) contained Tyrosine (Y) and Cysteine (C) residues, respectively. At the 40th residue of the SBP domain, K and R residues were identified in G8 (LcSPL7 and LtSPL7) and G2 (LcSPL2 and LtSPL2, LcSPL11 and LtSPL11) members, respectively. Additionally, a G residue was found at the 15th residue in G8 members (LcSPL7 and LtSPL7), while the other residues were Serine (S).

### Syntenic analysis of *SPLs* in *Liriodendron*

**Figure 4.**
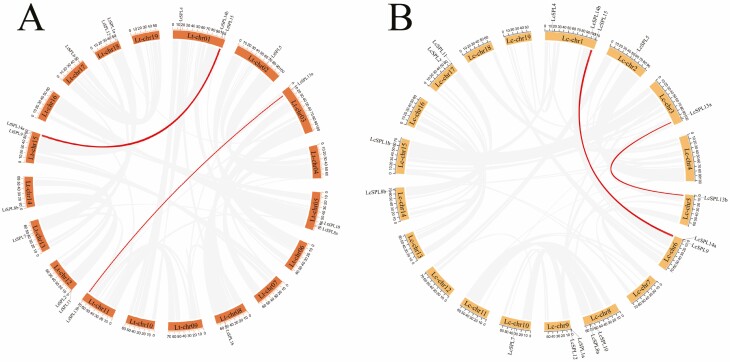
Intraspecific synteny analysis of *LcSPLs* and *LtSPL*. (A) Intraspecific synteny analysis of *SPL* genes in *L. tulipifera*. The chromatic line indicates there is a collinearity between two given *LtSPL* genes. (B) Intraspecific synteny analysis of *SPL* genes in *L. chinense*. The chromatic  line indicates there is a collinearity between two given *LcSPL* genes.

**Figure 5.**
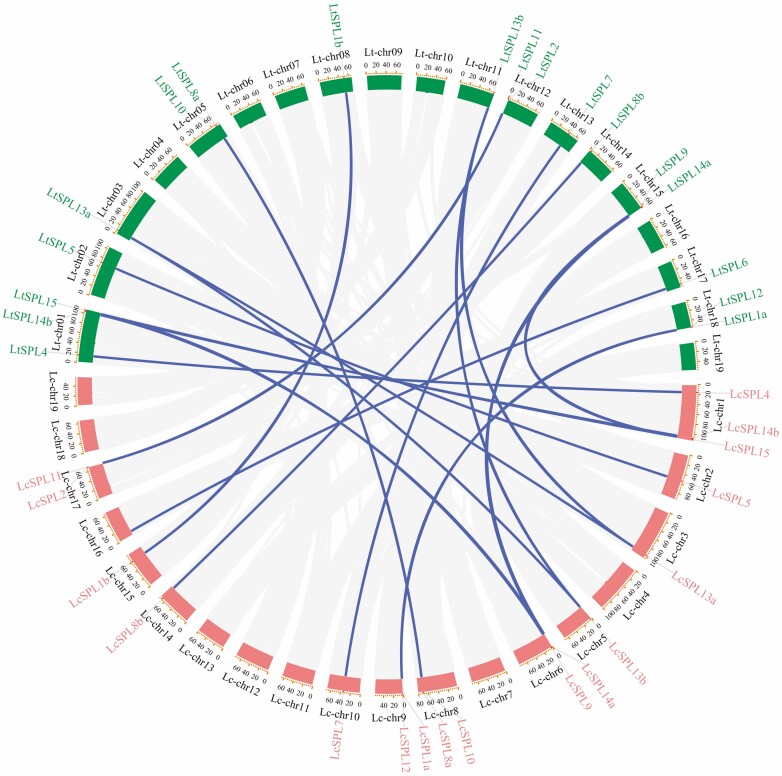
Synteny analysis of *SPL* genes from *LcSPLs* and *LtSPLs*. Grey lines in the background indicate collinear blocks between *L. chinense* and *L. tulipifera*, whereas the chromatic lines highlight syntenic *SPL* gene pairs.

The chromosomal location plot of the 35 *SPL* genes in *Liriodendron* revealed a non-uniform distribution across the 19 chromosomes (Fig. 4). Notably, both *LcSPLs* and *LtSPLs* showed segmental duplicates, with three pairs of duplicated genes identified in each species (*LcSPL9* and *LcSPL15*, *LcSPL13a* and *LcSPL13b*, *LcSPL14a* and *LcSPL14b* for *L. chinense*; *LtSPL9* and *LtSPL15*, *LtSPL13a* and *LtSPL13b*, *LtSPL14a* and *LtSPL14b* for *L. tulipifera*). However, no tandem duplications were observed in either species. Additionally, syntenic analysis indicated that, except for *LtSPL6*, all 17 *LtSPLs* exhibited collinear relationships with the *LcSPLs* (Fig. 5). The absence of syntenic homology for *LtSPL6* with *L. chinense* might be attributed to incomplete assembly of the *L. chinense* genome.

**Figure 6.**
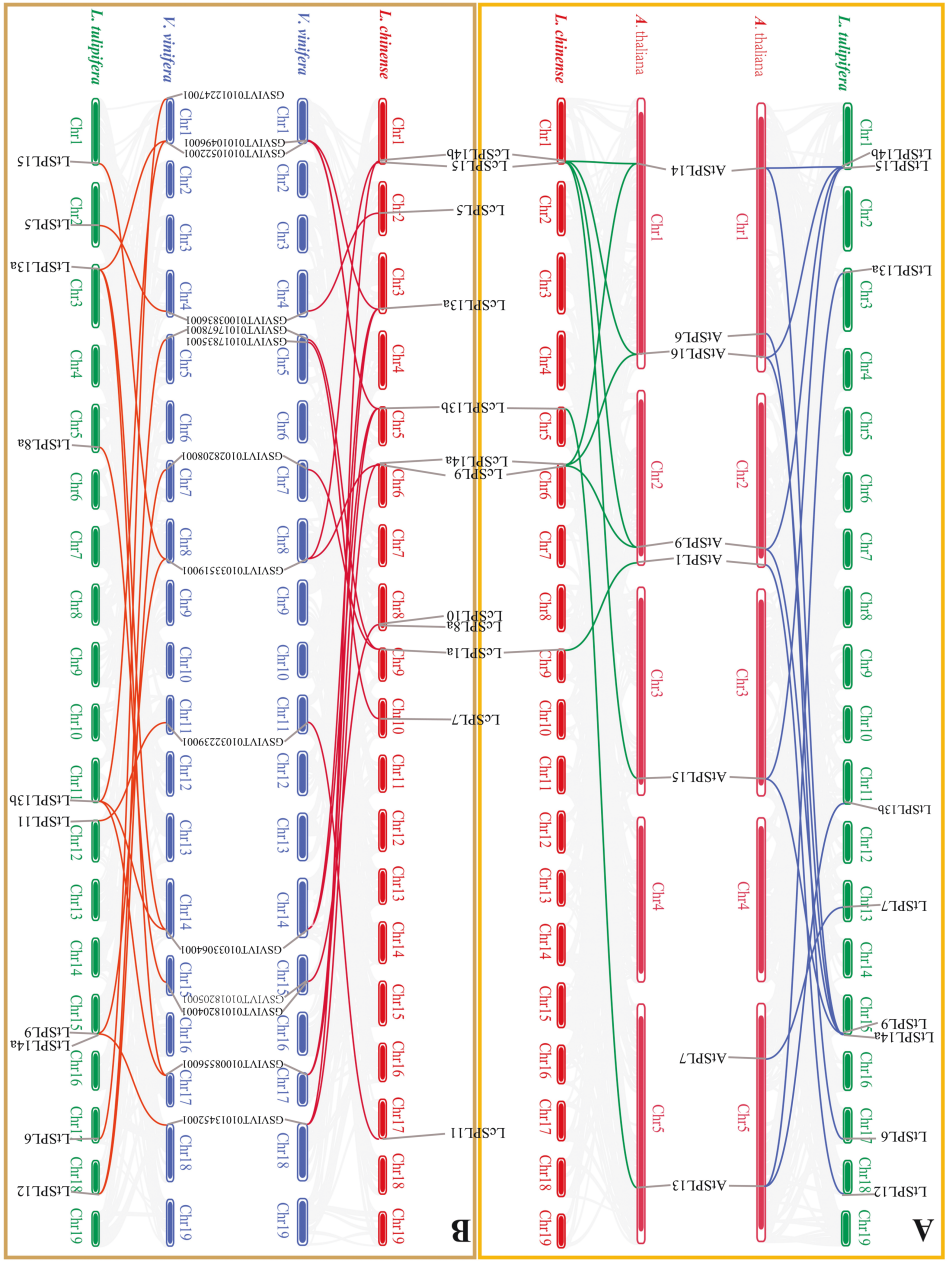
Synteny analysis of *SPL* genes among *L. chinense*, *L. tulipifera*, *A. thaliana* and *V. vinifera*. Grey lines in the background indicate the collinear blocks within different plant genomes, while colour lines highlight the syntenic *SPL* gene pairs.

In addition, to infer the evolutionary relationships of *SPLs*, we conducted synteny analysis across four dicotyledonous plants: *L. chinense*, *L. tulipifera*, *A. thaliana* and *Vitis vinifera*. The findings revealed homology between *SPL* genes in *Liriodendron* and those in *A. thaliana* and *V. vinifera*. In particular, the highest level of collinearity was observed between *L. chinense* and *V. vinifera*, with a total of 12 pairs of genes exhibiting collinear relationships (Fig. 6). Notably, *LtSPL6* exhibited collinear relationships with *V. vinifera* but not with *A. thaliana*. Overall, our results indicate a closer evolutionary affinity between *SPLs* from *Liriodendron* and *V. vinifera* compared with *A. thaliana*. This suggests that *SPLs* share a more recent common ancestor *V. vinifera* than with *A. thaliana*. These results support the notion that the *SPLs* may have evolved from a common ancestor shared by these different plant species.

### Identification of *cis*‑acting element of *LcSPLs* and *LtSPLs*

**Figure 7.**
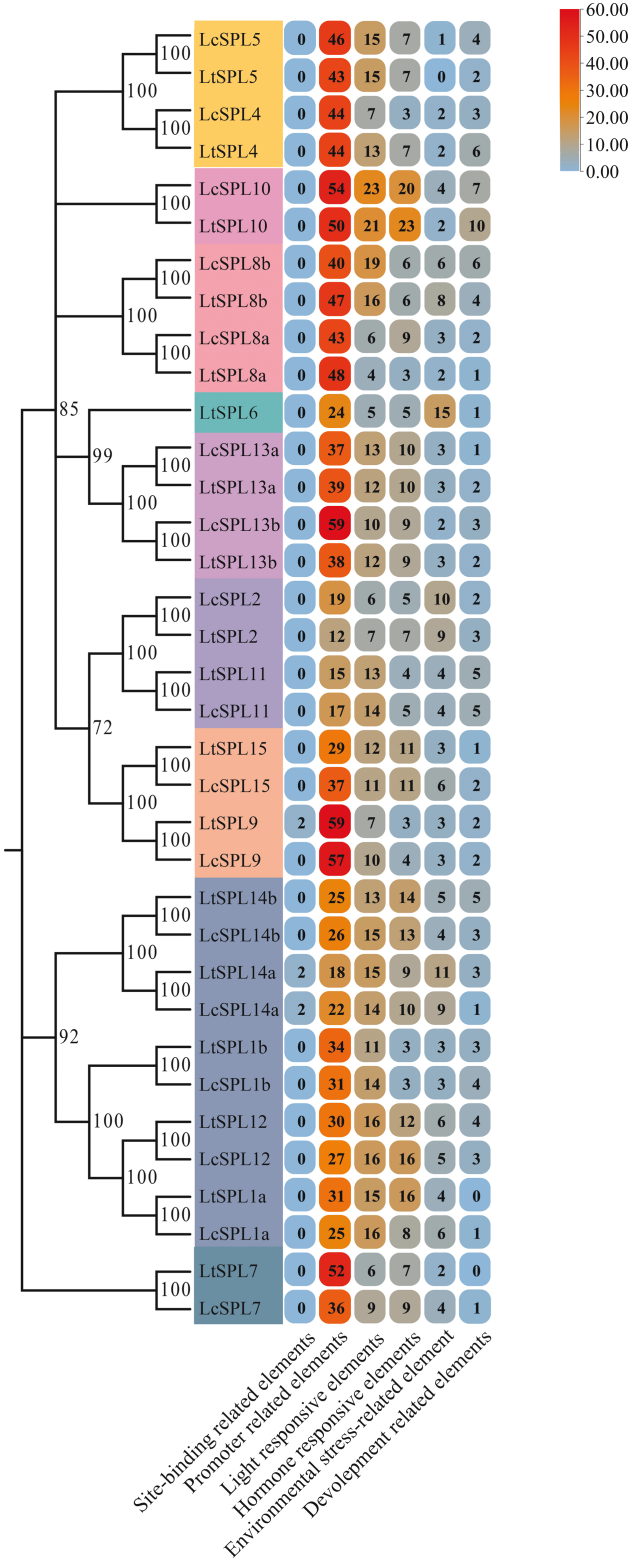
*Cis*-element regions of *SPL* genes promoters were analysed by PlantCARE in *L. chinense* and *L. tulipifera*. The numbers in the box represent the number of cis-element.

To elucidate the mechanisms of transcriptional regulation, we conducted an analysis of the *cis*-acting elements present in the promoters of *LcSPLs* and *LtSPLs*. A total of 57 types of *cis*-elements were identified from the 35 *SPL* genes in *Liriodendron* (Table 1). Based on their putative functions, these elements were categorized into eight groups: site-binding-related elements, promoter-related elements, light-responsive elements, hormone-responsive elements, environmental stress-related elements and development-related elements (Fig. 7).

The most prevalent *cis*-elements identified were the TATA box and CAAT box, both of which were present in all *LcSPLs* and *LtSPLs*. Moreover, light-responsive elements constituted the second largest group and were found in similar numbers as hormone-responsive elements within the promoter regions of all *SPL* genes. This noteworthy observation suggests that both *LcSPLs* and *LtSPLs* are intricately regulated in response to diverse environmental and developmental cues. Consequently, these findings reinforce the essential roles of *LcSPLs* and *LtSPLs* in governing critical physiological processes and coordinating complex developmental programs. Importantly, the distribution patterns of promoter elements in both *SPL* gene clades mirrored their homology distribution, underscoring the significance of these elements in gene regulation while highlighting their evolutionary conservation.

### Expression patterns of the *LcSPL* genes in different *L. chinense* tissues

**Figure 8.**
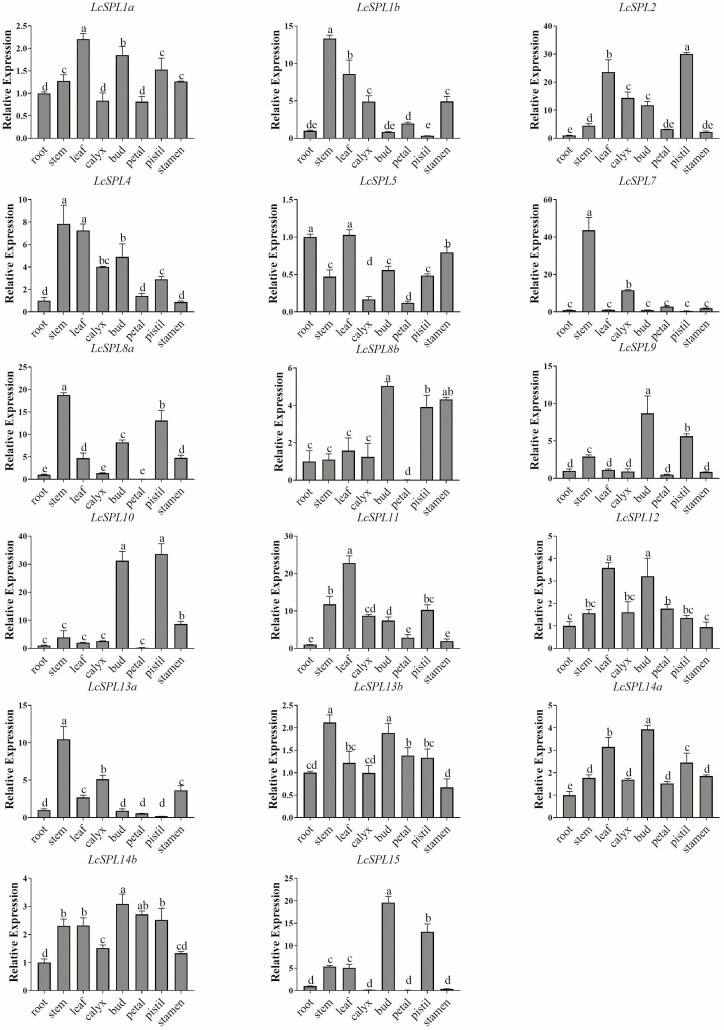
Expression analysis of *LcSPLs* in root, stem, leaf, calyx, floral bud, petal, pistil and stamen of *L. chinense*. The expression was determined by quantitative RT-PCR. *LcActin97* was used as an internal control. Means with different letters were significantly different. The error bars indicated ± SD of three technical replicates. Analysis of variance (ANOVA) with a subsequent Duncan’s test was performed (*P* < 0.05).

The distinct functions of genes in various plant tissues are often revealed through their unique expression patterns. To gain insights into the developmental roles of *LcSPLs*, we quantified the expression levels of the 17 *LcSPLs* across 8 tissues/organs of *L. chinense*, including root, stem, leaf, calyx, floral bud, petal, pistil and stamen (Fig. 8). Our RT-qPCR results unveiled diverse expression patterns of *LcSPLs* among these tissues/organs, with specific genes exhibiting tissue-specific expression profiles. Notably, homologous genes *LcSPL9* and *LcSPL10* were highly expressed in flower buds and pistils. Furthermore, *LcSPL2* showed the highest relative expression in pistils, with levels 30 times higher than those in the root, followed by leaves with a 24-fold increase, and approximately 15-fold higher in the sepal and flower bud compared with the root. Additionally, *LcSPL7* exhibited significantly higher expression in stem tips and sepals than in other tissues. Interestingly, several *LcSPL* members, including *LcSPL2*, *LcSPL9*, *LcSPL15*, *LcSPL10*, *LcSPL12*, *LcSPL14a*, *LcSPL14b* and *LcSPL8b*, demonstrated relatively high expression levels in floral buds and pistils. These findings illuminate the tissue-specific roles of *LcSPLs* and provide valuable insights into their potential functions in various developmental processes in *L. chinense*.

### Ectopic expression of *LcSPL2* in *A. thaliana*

**Figure 9.**
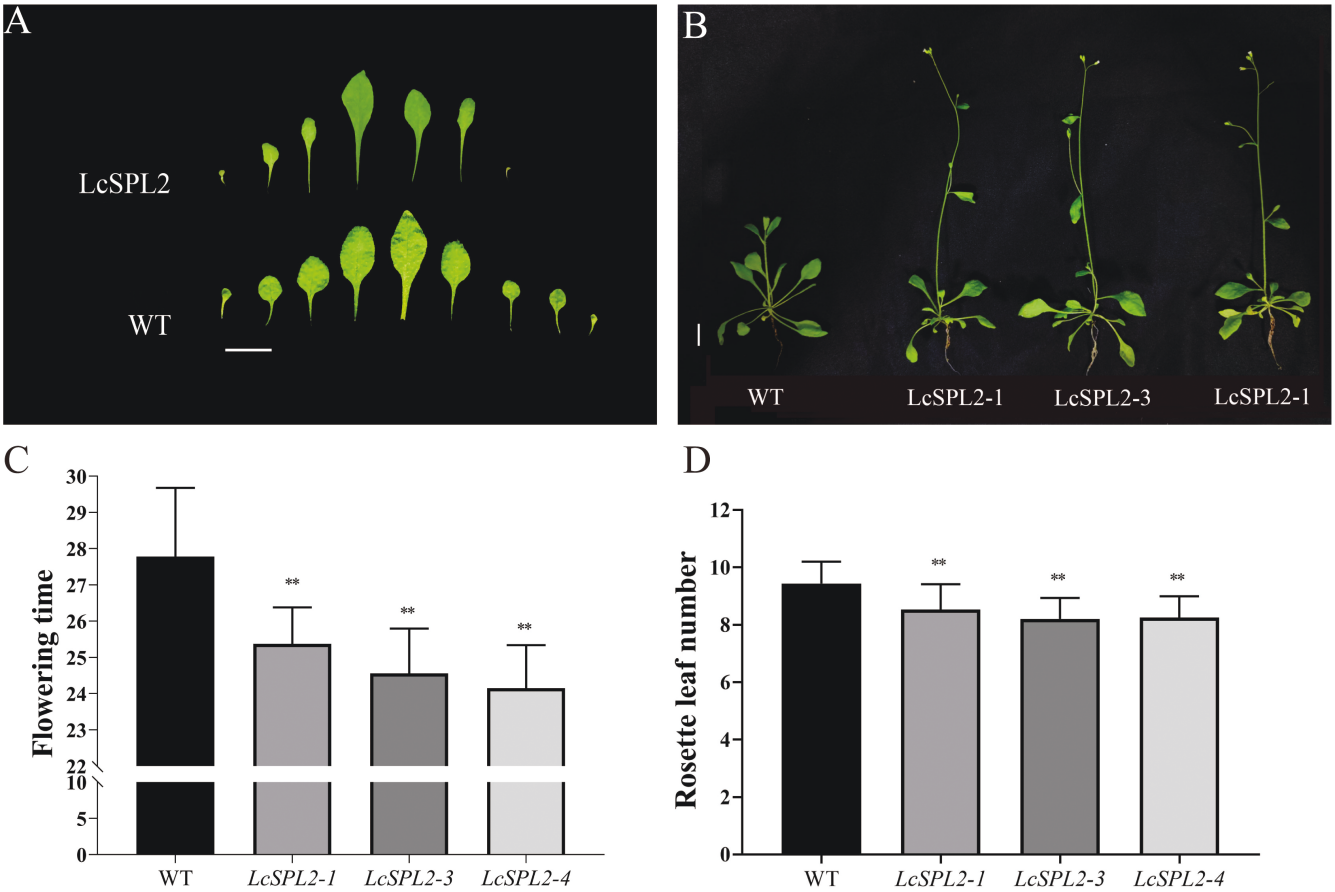
The phenotypes and relative expression levels of *LcSPL2* in the transgenic overexpression lines and WT *A. thaliana*. (A) The rosette leaf of WT *A. thaliana* and *35S::LcSPL2-1 A. thaliana*, bar = 1 cm. (B) The right three *A. thaliana* plants represented three transgenic lines, *LcSPL2-1*, *LcSPL2-3* and *LcSPL2-4*, bar = 1 cm. (C) Comparison of the number of rosette leaf at flowering between each *35S::LcSPL2* line and WT *A. thaliana*. (D) Comparison of the flowering time in each *35S::LcSPL2* transgenic line and WT *A. thaliana*. The first flowering duration was recorded in days from seeding to the occurrence of the first flower, unit in days. The error bars indicate the standard deviation (SD) of three biological replicates consisting of independent samples (no less than 10 plants per replicate). ANOVA with a subsequent Duncan’s test was performed (double asterisks, *P* < 0.01).

To investigate the impact of *LcSPL2* on flowering, we introduced its ectopic expression under the 35S CaMV promoter (*35S::LcSPL2*) in *A. thaliana* Columbia ecotype (Col-0) (WT), as depicted in Fig. 9. The base sequence of LcSPL2 can be found in Supplementary Material 1. We measured the time taken from seed sowing to the first flowering of the *35S::LcSPL2* transgenic plants under long-day (LD) conditions. Statistical analysis revealed a significant difference in the first flowering time between *35S::LcSPL2* and WT *A. thaliana*, with the *35S::LcSPL2* plants flowering significantly earlier. Furthermore, a reduction in the number of rosette leaves was observed in *35S::LcSPL2* compared with WT *A. thaliana* (Fig. 9).

**Figure 10.**
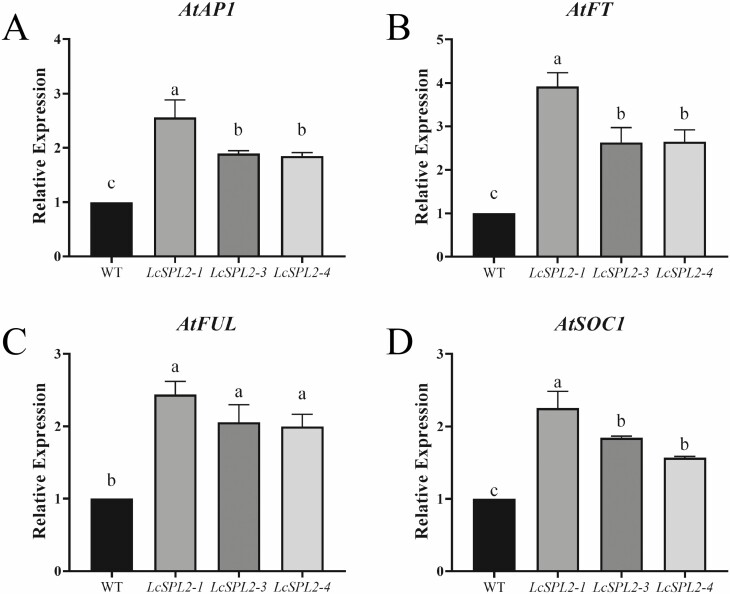
RT-qPCR analysis of four genes in WT *A. thaliana* and three *35S::LcSPL2* overexpression lines under LD condition. (A)–(D) RT-qPCR analysis of four flowering-related genes, *AtSOC1*, *AtFT*, *AtFUL* and *AtAP1*, in WT and transgenic *A. thaliana*. Samples were collected on the days to the initial flowering of *35S::LcSPL2* and WT *A. thaliana*. *AtActin2* was used as an internal control. The error bars indicate ± SE of three technical replicates. Means with different letters were significantly different. ANOVA with a subsequent Duncan’s test was performed (*P* < 0.05).

To investigate whether the expression of *LcSPL2* in the *35S::LcSPL2* transgenic *A. thaliana* influenced the expression of flowering-related genes, we performed RT-qPCR assays when the transgenic *A. thaliana* bloomed its first flower. Comparative analysis with WT plants revealed a significant increase in the expression of *AtSOC1*, *AtFT*, *AtFUL* and *AtAP1* genes in the *35S::LcSPL2* transgenic plants (Fig. 10).

These results suggest that the ectopic expression of *LcSPL2* in *A. thaliana* accelerates flowering and influences the expression of key flowering-related genes, providing valuable insights into the potential regulatory role of *LcSPL2* in the flowering process.

## Discussion

*SPL* genes are plant-specific TFs found across a wide range of green plants, including monocotyledonous, dicotyledonous and algal species (Cardon *et al.* 1999; Lannenpaa *et al.* 2004; Riese *et al.* 2008; Yang *et al.* 2008; Salinas *et al.* 2012). These genes play pivotal roles in regulating plant flower development and are involved in various plant growth and developmental processes, particularly in flowering regulation (Zhang *et al.* 2015; Han *et al.* 2016; Pan *et al.* 2017). In this study, we conducted a genome-wide analysis of the *SPL* genes in two *Liriodendron* species, *L. chinense* and *L. tulipifera*. Generally, *SPL* genes are classified into six to nine clades (Preston and Hileman 2013). The phylogenetic tree in this study showed that *LcSPLs* and *LtSPLs* clustered into nine clades. Paired homologous pairs of *L. chinense* and *L. tulipifera SPLs* were observed in each clade, with the exception of the G1 *LtSPL6* of *L. tulipifera*. Furthermore, we found that the *LcSPLs* of *L. chinense* exhibited a high degree of covariance with the *LtSPLs* in *L. tulipifera*. However, both species *SPLs* lacked tandem duplications. Previous studies have reported whole-genome duplication events in *Liriodendron* (Chen *et al.* 2018), suggesting that the differentiation of *SPL* genes in *Liriodendron* occurred before the divergence of *L. chinense* and *L. tulipifera*. Furthermore, we found that all groups contained SPL proteins from both monocots and dicots, indicating that *SPLs* existed prior to the divergence of dicots and monocots and subsequently evolved independently.

The SBP structural domains of both *LcSPLs* and *LtSPLs* exhibit highly conserved sequences, including CQQC, SCR, NLS, Zn-1 and Zn-2. This conservation is consistent with other plant species (Yang *et al.* 2008; Zhu *et al.* 2020). A total of 10 conserved motifs were identified among the SPL proteins. Interestingly, all 10 motifs were present in the G5 group, which also includes *AtSPL1/12* and exhibits similar motif distribution in tobacco (De Paola *et al.* 2023). Considering the high degree of sequence homology, along with similar conserved structural domain features and motif distributions among the same group of *LcSPL*s, these genes classified in the same group may have similar biological functions.

To gain further insights into the functions of *SPL* genes in *L. chinense*, we examined the expression levels of 17 *LcSPLs* in eight tissues. Generally, *SPLs* within the same clade share similar functions. Previous studies have indicated that *SPL* genes located in the same clade are involved in regulating flowering time in various plants like *A. thaliana*, rice and alfalfa (*Medicago sativa*) (Martin *et al.* 2010; Wang *et al.* 2012; Gao *et al.* 2018*b*). Notably, *AtSPL9/15* plays a crucial role in phase transition, anthocyanin accumulation and flowering regulation (Wang *et al.* 2009; Gou *et al.* 2011). In this study, we observed collinear relationships between *LcSPL9/15* and *AtSPL9/15*. Moreover, *LcSPL9/15* exhibited significantly elevated expression levels in flower buds and pistils. Therefore, further investigations are warranted to unravel the regulatory function of *LcSPL9/15* in flowering control in *L. chinense*. Understanding the role of *LcSPL9/15* could provide valuable insights into the intricate mechanisms governing flowering regulation in this species. Additionally, both *LcSPL2/11* genes showed high expression in leaves and pistils. Interestingly, three *AtSPL* genes (*AtSPL2/10/11*), from the same group as *LcSPL2/11*, have been previously identified as regulators of flowering time in *A. thaliana* (Yao *et al.* 2019; Ma *et al.* 2020). This finding indicates that *LcSPL2/11* may also be involved in the regulation of flowering time in *L. chinense*. Further investigations are needed to elucidate the specific roles of *LcSPL2/11* in the flowering process of this species.

Previous studies have conducted functional overexpression or deletion experiments on *SPL* genes in various plant species. For instance, in *Fortunella hindsii*, overexpression of three *FhSPLs* in *A. thaliana* resulted in early flowering phenotypes (Li *et al.* 2023). Similarly, the *AtSPL3/4/5* homologs from *Platanus acerifolia* induced early flowering when expressed in *A. thaliana* (Han *et al.* 2016). In alfalfa, *MsSPL13* and *MsSPL20* were found to regulate the vegetative growth phase. Plants overexpressing these *SPL* genes exhibit increased biomass and delayed flowering time (Gao *et al.* 2018*b*; Ma *et al.* 2022). In our study, we employed an ectopic expression system to investigate the function of *LcSPL2*. The transgenic *A. thaliana* plants with *35S::LcSPL2* showed an early-flowering phenotype (Fig. 8), with no significant changes in flower morphology, as anticipated.

As the primary effectors in the age-flowering pathway, *SPL* genes play a crucial role in regulating flowering time through two main mechanisms (Teotia and Tang 2015). Firstly, they operate within the microRNA regulatory network by activating downstream miR172, which, in turn, inhibits the activity of the *AP2s* and releases the activity of *SOC1* and other genes to promote flowering (Yant *et al.* 2010). Secondly, *SPL* genes directly bind to the promoters of *FT*, *SOC1*, *AP1* and *FUL*, leading to the promotion of flowering in plants (Wang *et al.* 2009; Yamaguchi *et al.* 2009; Kim *et al.* 2012). In this study, we observed a significant increase in the expression of *AtSOC1*, *AtFT*, *AtFUL* and *AtAP1* in *35S::LcSPL2* transgenic plants. This suggests that ectopic-transformed *LcSPL2* promotes flowering by increasing the expression levels of flowering-related genes in *A. thaliana*. However, further investigation is needed to elucidate its regulatory mechanism with genes such as *FT* and *SOC1* in *L. chinense*. Additional studies will be required to unravel the complex regulatory network underlying flowering in *L. chinense*.

## Conclusion

This study comprehensively analyses the *SPL* gene family in *L. chinense* and *L. tulipifera*. Seventeen *LcSPL* genes and 18 *LtSPL* genes were identified and divided into 9 groups. Tissue expression analysis showed that *LcSPLs* were expressed more in flower-related tissues. Overexpression of *LcSPL2* in *A. thaliana* accelerates flowering under LD conditions. Our study lays a cornerstone for further understanding the functions of *LcSPL* genes.

## Supporting Information

The following additional information is available in the online version of this article –

Supplementary material 1. Sequence for *LcSPL2*.

Supplementary material 2. Primers for PCR.

plae008_suppl_Supplementary_Material_S2

plae008_suppl_Supplementary_Material_S1

## Data Availability

The data for the *Lirioderon chinense* and *Lirioderon tulipifera* used in the article are sourced from publicly available genomes of the respective species: (i) *L. chinense*: NCBI Taxonomy ID: 3414 and (ii) *L.tulipifera*: http://phytozome-next.jgi.doe.gov/.
