# Supplementary material for: Genome-wide investigation of SQUAMOSA promoter binding protein-like genes in Liriodendron and functional characterization of LcSPL2
Source: AoB Plants. 2024 Feb 20;16(2):plae008. doi: 10.1093/aobpla/plae008 (PMC10908533; doi:10.1093/aobpla/plae008)
Supplement: plae008_suppl_Supplementary_Material_S1 [file plae008_suppl_supplementary_material_s1.docx]

>LcSPL2

ATGGATTCTTCGTCAAAGACTGGACTCAAGGATTTCGAGTATAATCTTCAGACAGCCAAAGGTTTTCCTAAAGATCTTAACAAAAAGAAAGAATTGGGTAGGGTGGAAGACACTGGAACTTCTCCAGAAATTTTGGCATCTATTGGCAATGGAGAGCCTGTGATTGGTCTAAAGCTTGGAAAGCGAACTTACTTTGAAGATGTTGGTGCTGGGAGCACTGTTAAGACAACATCATCTAGTTCTGCGATTTCCACATCATCTAACAATTCTATTAAGAAGTCAAGGGCATCATATCAGAGCATGCAGGTCCCTCGCTGCCAGGTCGAAGATGGCAGGCTTGTGTTGAACAGGCTTCCACTTATTCATGAAAGACATGCTGTGAATACTGCACGGGAGAGCTCACATGACTTCAAGATTGCACAAGCAAAAGGGTCTTGGATAAGGACTGTGAAAACAGGAGGCATTGATGGTCATCTGCATTTGCCAAGTGCTGATCTGCCAACTACCATCCCCATCCTTTCTCATGACTTTGACAAGCTATTGCCATTCAAGGGCACCTCTGCCGGGGTCCTCAATCAAGGTGCGGTTAACTTCCTCTGTTTTATTTCTGGTCAGGAAGCATCTATGATTGCTTCCAACTCAGATACAACACTGGACTCTCGGCGTGCTCTCTCTCTTCTGTCAACTAACTCATGGGGTTTGGTTCACCCCGAACCAACTTCTCTCGACCAGCTCATGCATGCAAACAATGCAAGCATGGGTCAGCCAGCAATGGCCCCAGTAGATCAAGTTCTGCCACATGCTAGCCCTGACTACTGGCAGGCCGAACTGCGAGCGCCACCATTGACCTTGCATAGCACTGGTGGTCAGTTCCAAGAGTTTCAGCTTTTCAAAGCACCCTATGAATCTGCCTTTCTCAACAGCAACCATATGCACTGA
